# Implantation of ^125^I radioactive seeds via c-TBNA combined with chemotherapy in an advanced non-small-cell lung carcinoma patient

**DOI:** 10.1186/s12890-019-0974-8

**Published:** 2019-11-08

**Authors:** Ai-Gui Jiang, Hui-Yu Lu, Zhong-Qi Ding

**Affiliations:** 1 Department of Respiratory and Critical Care Medicine, Taizhou People’s Hospital, 210 Yingchun Road, Taizhou, Jiangsu 225300 People’s Republic of China; 2 Department of Nuclear Medicine, Taizhou People’s Hospital, 210 Yingchun Road, Taizhou, Jiangsu 225300 People’s Republic of China

**Keywords:** Non-small-cell lung carcinoma, ^125^I radioactive seed, Transbronchial needle aspiration

## Abstract

**Background:**

The critical management of advanced non-small-cell lung carcinoma (NSCLC), especially when complicated by severe airway stenosis, is difficult and often leads to high clinical risks and medical costs.

**Case presentation:**

A 51-year-old previously healthy male was admitted to the Department of Respiratory and Critical Care Medicine, Taizhou People’s Hospital, in November 2018 for haemoptysis and difficulty breathing during a 15-d period. Following admission, chest computed tomography (CT) showed a large mass in the left hilum with atelectasis in the left upper lobe and obstructive pneumonia in the left lower lobe. Bronchoscopy revealed that the lesions occurred in the distal segment of the left main trachea, with occlusion of the left upper bronchus and significant narrowing of the lower bronchus. A basal mucosal biopsy of the lump tissue was performed after haemostasis treatment with sub-plasma coagulation (APC), and squamous lung carcinoma was confirmed. Following the final diagnosis, the patient was successfully treated with implantation of ^125^I radioactive seeds via transbronchial needle aspiration (c-TBNA) combined with chemotherapy.

**Conclusion:**

We believe that implantation of ^125^I radioactive seeds via c-TBNA is an effective treatment for patients with advanced lung cancer and those presenting with severe and mixed main bronchus stenosis.

## Background

Non-small-cell lung carcinoma (NSCLC) is the most common type of cancer worldwide. Almost 70–75% of patients with NSCLC have advanced stage IIIB or IV disease when first diagnosed [1]. Advanced NSCLC, particularly at the time where the central airway is involved, which often leads to severe airway stenosis, is a dangerous disease that is challenging to manage and has a poor prognosis [2]. Although substantial progress has been made in terms of bronchoscopy interventional techniques, such as electrocoagulation, sub-plasma coagulation (APC), airway stenting, laser therapy and balloon dilatation in recent years [3], the critical management of some patients with airway stenosis is difficult and remains a focus of attention. The choice of therapy depends on the type of airway stenosis, pulmonary function and life expectancy of the patient. In recent years, studies have examined ^125^I radioactive seed implantation performed under CT or ultrasound guidance for the treatment of lung cancer, and many patients have been reported to benefit from this alternative technique [4]. However, central bronchogenic carcinoma patients cannot benefit from this alternative technique because of a high risk of haemorrhage or pneumothorax [5, 6]. To the best of our knowledge, reports of ^125^I radioactive seed implantation via transbronchial needle aspiration (c-TBNA) are rare [7]. The present study reports the diagnosis of an advanced NSCLC patient with severe airway stenosis and the outcomes of treatment with ^125^I radioactive seed implantation combined with chemotherapy.

## Case presentation

This case report was carried out in accordance with the Declaration of Helsinki and was approved by the Ethics Committee of Taizhou People’s Hospital, Jiangsu, China. Written informed consent was obtained from the patient. A 51-year-old previously healthy male was admitted to the Department of Respiratory and Critical Care Medicine, Taizhou People’s Hospital, in November 2018 for haemoptysis and difficulty breathing during a 15-d period. Following admission, physical examinations revealed that vital signs were stable. Left-side respiratory movement was weakened and left upper lung tremor was significantly enhanced. Expiratory wheezing was heard throughout the left lung, and no moist rales were noted. The patient had a regular heart rhythm, and no oedema was observed in the lower extremities.

**Fig. 1:**
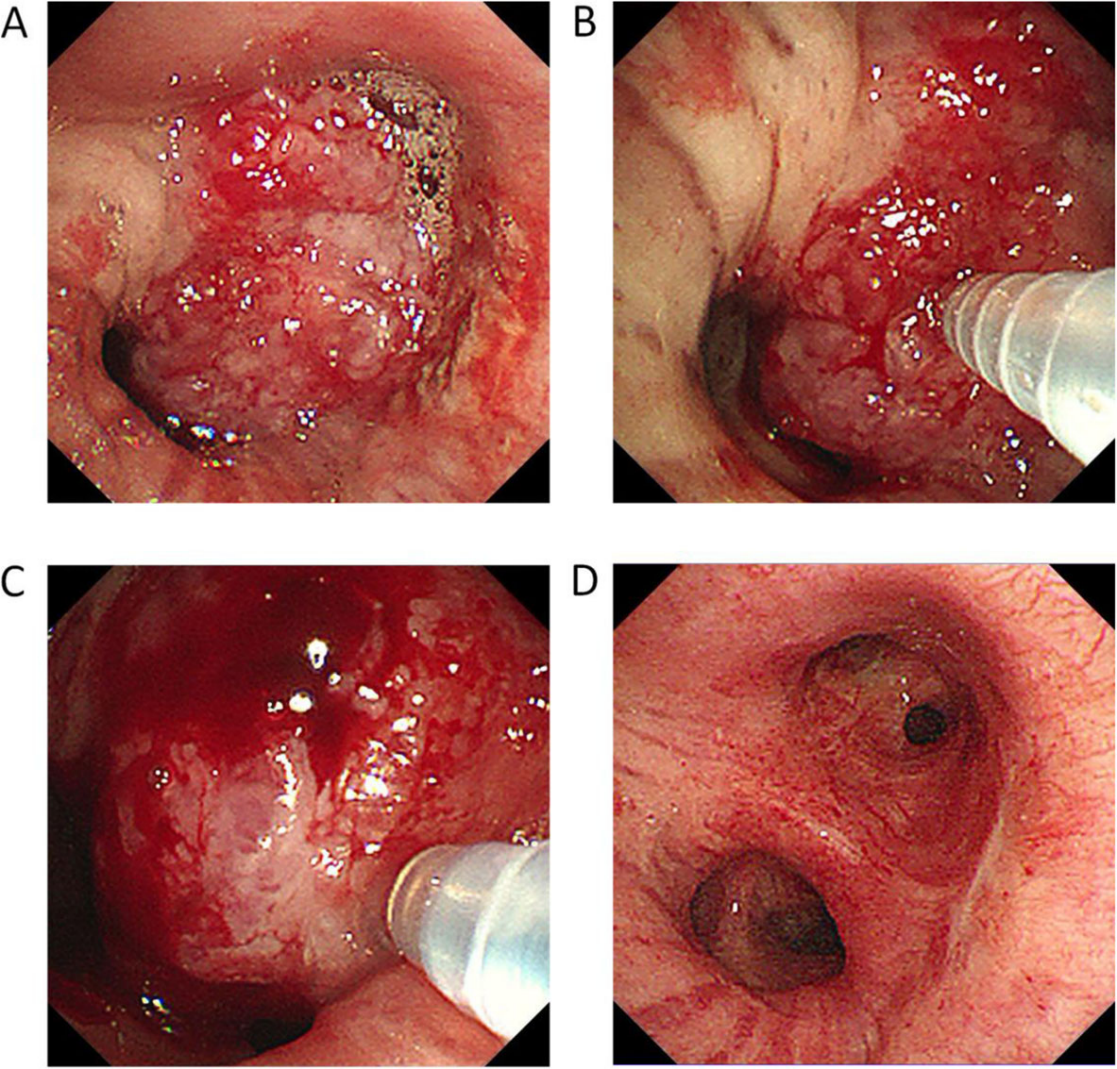
**a** Bronchoscopy revealed that the lesions occurred in the distal segment of the left main trachea, with occlusion of the left upper bronchus and significant narrowing of the lower bronchus. **b**, **c** ^125^I radioactive seeds were pushed into the tumour via the core of the needle after the puncture needle was passed through the wall of the externally compressed trachea or into the tumour. **d** Bronchoscopy at the second cycle after chemotherapy revealed disappearance of the lesions in the distal segment of the left main trachea, with no obstruction of the left upper lobe or the left lower lobe

A complete blood test revealed a white blood cell count of 6.84 × 10^9^/l and a large white blood cell ratio (percentage of white blood cells that are neutrophils) of 82.8%. A blood gas analysis demonstrated a pH of 7.38, 90 mmHg PaO_2_, 35 mmHg PaCO_2_ and 29 mmol/l HCO_3_^−^. Serum tumour markers demonstrated 3.56 ng/ml of CEA, 18.75 ng/ml of Cyfra21–1 and 1.2 ng/ml of SCC. No obvious abnormalities in liver or kidney function were observed. CT showed 4.1 × 7.1 cm lesions with irregular, thick-walled, hollow and unclear boundaries in the left hilum, as well as atelectasis in the left upper lobe, obstructive pneumonia in the left lower lobe and a mass in the mediastinum and left hilar lymph node. Bronchoscopy revealed that the lesions occurred in the distal segment of the left main trachea, with occlusion of the left upper bronchus and significant narrowing of the lower bronchus (Fig. 1a). A basal mucosal biopsy of the lump tissue was performed after haemostasis treatment with APC, and squamous lung carcinoma was confirmed. The patient was eventually diagnosed with left upper lung squamous cell carcinoma, with stage IIIB (cT_3_N_2_M_0_) and ECOG’s PS1.

**Fig. 2:**
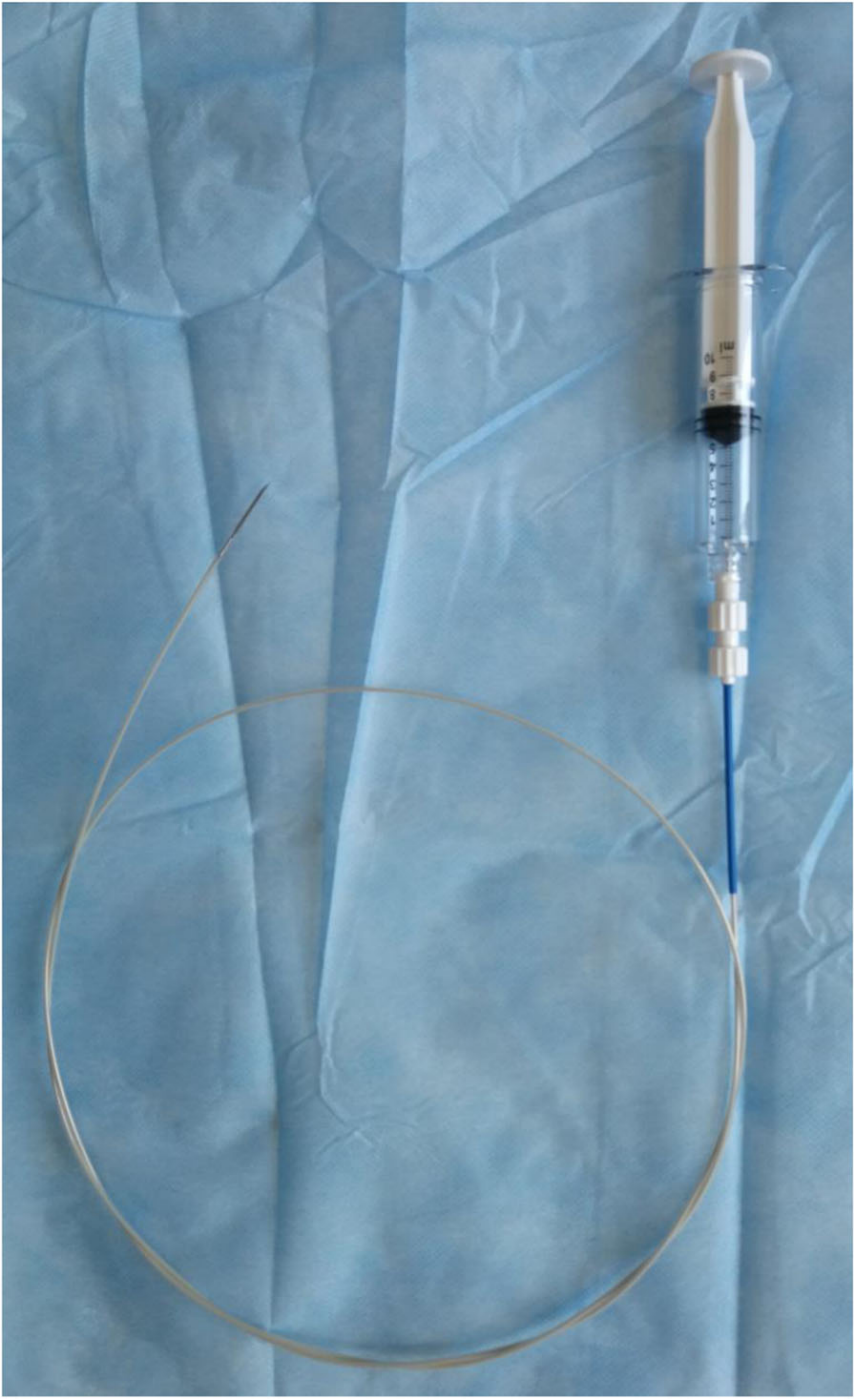
Endoscope biopsy sampling needles: The inner tube of the tissue needles consisted of a hose with a steel wire directly connected to the syringe, which could be automatically moved during suction. 2–3 ^125^I radioactive seeds were placed in the biopsy channel of the endoscope biopsy sampling needles after suction of the syringe to the specified scale. The channel port of the needles was blocked with sterile glucose agar after the placement of the seeds. ^125^I radioactive seeds were pushed into the tumour by using the steel wire

**Fig. 3:**
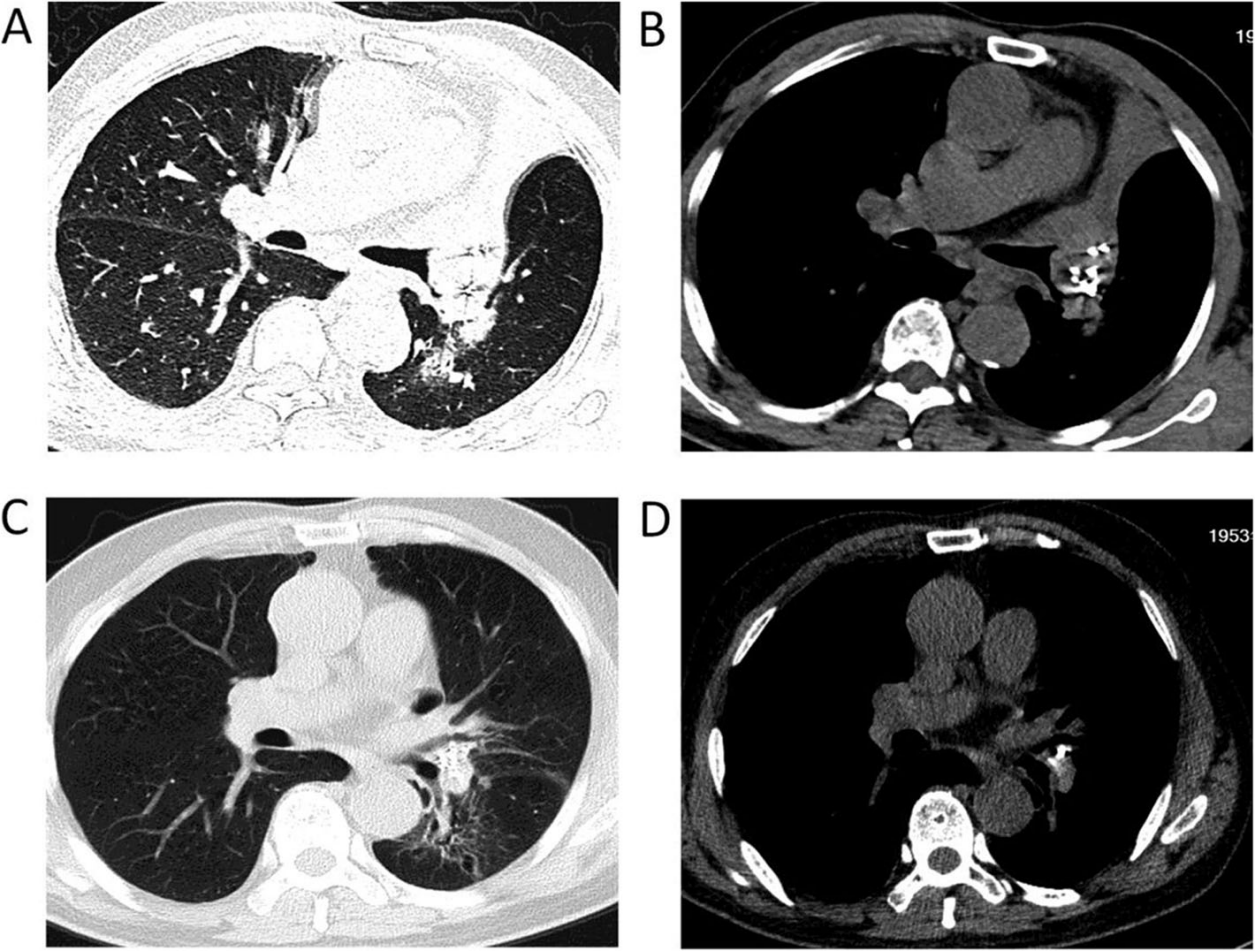
**a**, **b** Post-operative CT 3 days later confirmed that high-density ^125^I radioactive seeds were observed in the tumour (**a**: lung window, **b**: mediastinal window). **c**, **d** Compared with the previous chest CT scan, the chest CT scan at the second cycle after chemotherapy revealed disappearance of atelectasis and obstructive pneumonia in the left upper lung, as well as evident improvements in the lesions of the left hilum (**a**: lung window, **b**: mediastinal window)

Following confirmation, the patient was treated with ^125^I radioactive seed implantation via c-TBNA combined with chemotherapy. The implantation of 0.7 mCi ^125^I radioactive seeds (catalogue no., CIAE-6711; Chinese Atomic Energy Science Institution, Beijing, China) was used for this patient. The dose of the radioactive seeds was determined according to the following empirical formula: total dose (mCi) = (length + width + height) / 3 × 5. The number of seeds was calculated as follows: number of seeds = total dose/0.7. The prescription dose determined from this formula was expected to reach 100–130 Gy [7]. The endoscope was obtained from Olympus (BF-1 T260; Olympus Corporation, Tokyo, Japan). The technique used for ^125^I radioactive seed implantation was, overall, the same as that used for the regular c-TBNA examination and was performed under general anaesthesia of the airway with the use of a laryngeal mask and monitoring of the heart (via an electrocardiogram), pulse, blood pressure and blood oxygen saturation. Prior to implantation, 2–3 seeds were placed in the biopsy channel of the endoscope biopsy sampling needles (Changzhou Detan Medical Devices Co., Ltd., Changzhou, Jiangsu, China) (Fig. 2). The channel port was blocked with sterile glucose agar after the placement of the seeds. ^125^I radioactive seeds were pushed into the tumour via the core of the needle after the puncture needle passed through the wall of the externally compressed trachea or into the tumour (Fig. 1b, c). Based on chest CT and bronchoscopy, the size of the lesions in the left hilum was approximately 1.8*2.1*2.6. Eighteen seeds were implanted via c-TBNA in the patient. The distance between each puncture point was controlled at approximately 0.5 cm, and the puncture needle was placed perpendicular to the lesion during the puncture in order to ensure an even radiation dose in the lesion. To avoid unnecessary radiation pollution, the operation was carried out in the DSA operating room with adequate protective covering for the operators. The operation was successfully performed, and the vital signs of the patient were stable during and after the operation. Post-operative CT 3 days later confirmed that high-density ^125^I radioactive seeds were observed in the tumour (Fig. 3a, b). After the implantation of the ^125^I radioactive seeds, the patient received chemotherapy with GP (gemcitabine: 1000 mg/m^2^, i.v., days 1 and 8; cisplatin: 80 mg/m^2^, i.v., day 1), which was repeated every 21 days. At the first cycle after chemotherapy, the patient demonstrated a distinctly improved mental condition with no further activity haemoptysis attacks, as well as a distinct improvement of left chest tightness. Compared with the previous chest CT scan, a chest CT scan at the second cycle after chemotherapy revealed disappearance of atelectasis and obstructive pneumonia in the left upper lung, as well as evident improvement of the lesions in the left hilum (Fig. 3c, d). A follow-up bronchoscopy at the second cycle after chemotherapy revealed disappearance of the lesions in the distal segment of the left main trachea, with no obstruction of the left upper lobe or the left lower lobe (Fig. 1d). Following two rounds of chemotherapy, the patient’s disease was controlled, and partial remission of lung cancer was achieved.

## Discussion and conclusions

The critical management of advanced NSCLC, especially when complicated by severe airway stenosis, is difficult and often leads to high clinical risks and medical costs. Opening of the narrow trachea as soon as possible is one of the key factors affecting the prognosis of the disease [8]. Although there has been considerable progress in recent years in terms of the development of bronchoscopy interventional techniques, including electrocoagulation, APC, airway stenting, balloon dilatation, laser therapy and cryoablation, the critical management of some patients with obstruction of the distal section of the central airway caused by intraluminal tumour growth or extrinsic compression is difficult and remains a focus of attention [9].

Radioactive seeds are implanted directly into the lesion as a kind of brachytherapy, and these seeds kill the tumour cells by consistently releasing rays [10]. ^125^I radioactive seeds represent the most common type of permanently implanted seed, which can continuously release low-power γ-rays, with a half-life of 59.6 days and an available irradiation range of 17 mm, thus continuously inhibiting cell proliferation and angiogenesis as well as inducing apoptosis and killing tumour cells [11]. Percutaneous ^125^I radioactive seed implantation is usually performed under computed tomography (CT) or ultrasound guidance, and this technique has demonstrated effectiveness in local control and improvements of the OS time and rate [12–15]. However, for central-type lung cancer, the technique of percutaneous puncture is associated with a great risk of haemorrhage and pneumothorax [7].

With the development of endoscopic interventional diagnosis and treatment techniques, trans-bronchoscopy ^125^I radioactive seed implantation has been reported for the treatment of central airway stenosis [7]. This technique is mainly guided by bronchoscopy; ^125^I radioactive seeds are implanted into the tumour tissue via c-TBNA or EBUS-TBNA technology [16]. After implantation, the ^125^I radioactive seeds inhibit cell proliferation and angiogenesis, induce apoptosis, and kill tumour cells [11]. This technology provides a new and effective palliative treatment for patients with central lung cancer; it can permanently destroy tumour cells, maximize the patient’s lung function, improve the patient’s tracheal patency, and improve the patient’s quality of life.

The lesions in the reported patient occurred in the distal segment of the left main bronchus, with occlusion of the left upper bronchus and significant narrowing of the lower bronchus. To the best of our knowledge, traditional respiratory endoscopic intervention techniques, such as laser ablation and stent placement, which are considered the gold-standard treatments for narrowing of the trachea and main bronchus, were not effective and thus were not preferred methods for the specific site of bronchus narrowing in the presented patient. The site of bronchus narrowing was the real problem for the patient; thus, there was a need for a different type of therapy. Fortunately, the patient was successfully treated with trans-bronchoscopy with ^125^I radioactive seed implantation combined with chemotherapy. We believe that the treatment strategy used for this patient may have opened the respiratory tract in a short period of time, reduced the clinical symptoms, and thus improved the patient’s quality of life and survival time.

In conclusion, the majority of patients with advanced central lung cancer eventually require palliative treatment and individualized treatment strategies. Trans- bronchoscopy with ^125^I radioactive seed implantation combined with chemotherapy may be a potential and alternative treatment for some patients. It has been reported that 3 to 4 days after ^125^I radioactive seed implantation may be the best time for initial chemotherapy because the radioactive effect of the ^125^I radioactive seeds at this time result in an increase in vascular permeability and a facilitation of drug penetration [17]. However, due to the limitation of the number of patients, a mature and feasible radioactive particle-based comprehensive program for these patients still requires further large-scale clinical research.

## Data Availability

The datasets used and/or analyzed during the current study are available from the corresponding author on reasonable request.

## Abbreviations

APC: Sub-plasma coagulation
CT: Computed tomography
c-TBNA: transbronchial needle aspiration
NSCLC: Non-small-cell lung carcinoma
